# Refractory Temporomandibular Joint Dislocation – Reduction Using the Wrist Pivot Method

**DOI:** 10.5811/cpcem.2017.9.35834

**Published:** 2017-10-18

**Authors:** Vincent W.M. Lum, Juliana Poh

**Affiliations:** * Changi General Hospital, Department of Accident & Emergency, Singapore, Singapore; † Singapore General Hospital, Department of Emergency Medicine, Bukit Merah, Singapore

## Abstract

We report a case of a 19-year-old male who presented to the emergency department with refractory atraumatic temporomandibular joint dislocation. Multiple attempts at reduction by emergency physicians before and after sedation were unsuccessful. The dislocation was eventually reduced using the wrist pivot technique. This case highlights the need to consider alternative methods of temporomandibular joint reduction, particularly in cases refractory to reduction despite the use of sedation.

## INTRODUCTION

Temporomandibular joint (TMJ) dislocation is a condition that occurs when the mandibular condyle becomes displaced from the mandibular fossa. Anterior and bilateral dislocations are more common.^1^ It is frequently due to wide opening of the mouth such as during yawning, laughing or singing and may also occur during intubation, endoscopy or dental/ear nose throat (ENT) procedures.^2^ It may be a result of trauma^2^ as well. Following TMJ dislocation, the spasm of the masseter, temporalis and internal pterygoid muscles prevents return of the condyle to the mandibular fossa.^1^,^2^ This case report highlights the importance of considering alternative methods, such as the wrist pivot technique, in the management of patients with refractory TMJ dislocation.

## CASE REPORT

We present a case of a 19-year-old Chinese male who sustained refractory left TMJ dislocation while yawning in the early hours of the night. He presented to the emergency department (ED) within one hour of the dislocation. There was a history of TMJ dislocation, which had been reduced without sedation previously.

Eight different doctors, including a consultant emergency physician and two senior residents, attempted reduction multiple times unsuccessfully using the conventional technique. The patient was sedated and reduction re-attempted without success with the same technique. Radiographs were subsequently ordered, which confirmed the dislocation and did not reveal any fractures or other structural causes to account for the difficulty.

Finally, the wrist pivot technique was attempted while the patient was still sedated. The mandible was relocated on the first attempt using this technique. Minimal force was required to maneuver the dislocated portion back in place. It was easy to master, as it was the author’s first attempt using the wrist pivot technique, following only written instructions. The method was discovered while performing a literature search online after the multiple failed attempts using the conventional technique.

## DISCUSSION

There are multiple techniques for reducing TMJ dislocations.^1^ These include the conventional intraoral technique whereby the doctor stands facing the patient and inserts two thumbs wrapped with gauze onto the inferior molars with the rest of the fingers around the external mandible, then applying steady firm downward and backward pressure to relocate the jaw.^1^ This is also known as Nélaton’s maneuver or the Hippocratic technique.^3^ A variation of this technique has the doctor standing behind the patient instead, similarly inserting gauze-wrapped thumbs onto the inferior molars.^1^ Disadvantages of Nélaton’s maneuver include the force required to reduce the jaw as well as the risk of injury to the thumbs from the forceful contraction of the masseters upon successful reduction.^1^ And sedation is often required, with its potential risk.

An alternative method called the wrist pivot technique^4^ was described by Lowery et al. in 2004. This technique involves the physician grasping the mandible at the mentum with both thumbs and placing the fingers on the inferior molars, applying upward force on the thumbs and downward pressure with fingers. The wrist is then pivoted to reduce the dislocated jaw. According to the authors, the forces should be applied bilaterally to avoid mandibular fracture. In their case report, the patient was sedated initially for the attempt using the conventional method and at the time of using the wrist pivot method; the most recent sedation had been 20 minutes prior.

Another technique involving an extra-oral approach^5^,^6^ was described by Chen et al. in 2007 and subsequently used by Ardehali in 2009. It involves placing the thumb of one hand on the malar eminence of the maxilla, with the remaining fingers around the angle of the mandible. At the same time, the thumb of the other hand palpates the coronoid process on the contralateral side, with the remaining fingers posterior to the mastoid process. Once in position, the doctor then pulls the angle of the mandible anteriorly while simultaneously using the other hand to push the coronoid process posteriorly. This relocates the TMJ on the side of the coronoid process. Once one side is reduced, the other side usually returns spontaneously. In Chen’s case series of seven patients, none required sedation. In addition, the fact that this technique does not require placement of thumbs or fingers into the patient’s mouth reduces the risk of inadvertent injury to the physician from the patient’s teeth during reduction.

**CPC-EM Capsule**

What do we already know about this clinical entity?

Temporomandibular joint dislocation can be reduced by the conventional technique of downward and backward pressure on the lower molars.

What makes this presentation of disease reportable?

This temporomandibular joint dislocation was refractory to reduction despite multiple attempts under sedation using the conventional technique.

What is the major learning point?

Consider alternative techniques for reduction of refractory temporomandibular dislocations, such as the wrist pivot technique.

How might this improve emergency medicine practice?

This technique enables faster turnaround of patients in the ED for a distressing condition, while potentially avoiding the risks of sedation.

A more recent method, described by Gorchynski et al. in 2014, is known as the syringe technique.^7^ The patient is instructed to bite down on a 5ml or 10ml syringe between the molars on the affected side. He is then asked to roll the syringe to and fro between his teeth until relocation occurs. Neither sedation nor manual manipulation is required, unlike the other techniques described above. However, it does require the patient’s cooperation in understanding and complying with the instructions. Also, if the patient had been sedated prior to attempting this technique, it is necessary to allow him to regain consciousness first.

Stimulation of the gag reflex,^8^ the unified hands technique^9^ and manipulation for disk displacement^10^ have also been described as alternative methods.

As can be seen from the discussion above, multiple techniques^11^,^12^ exist for reduction of TMJ dislocations, each with its own pros and cons. No single technique has proven superior to other methods and can be used in all situations. The utility of some of these techniques in traumatic or non-anterior dislocations may vary as well. One possible way of approaching an acute atraumatic anterior dislocation might be to start with the syringe technique, followed by the extra-oral, then the wrist pivot method and finally the conventional approach with or without sedation.

Since this first attempt using the wrist pivot technique on the above patient with refractory TMJ dislocation, the author has used it on multiple other patients with good results and without sedation. There was even an instance whereby the dislocation was reduced with the patient still on the ambulance gurney, before being transferred to the hospital wheelchair. This technique has enabled faster turnaround of patients in the ED for a painful and distressing condition.

## CONCLUSION

It is advisable for emergency physicians to consider alternative techniques for reduction of refractory TMJ dislocations, such as the wrist pivot technique, especially in cases where sedation needs to be avoided. With other less traumatic methods available for reduction, perhaps the conventional technique should now be considered last.

## Figures and Tables

**Image 1:**
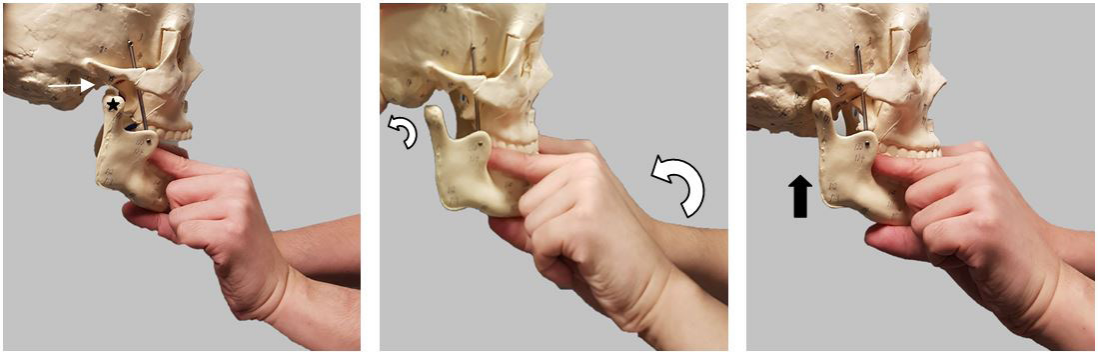
Wrist pivot technique. Empty mandibular fossa (thin white arrow), Dislocated mandibular condyle (black star). Wrist pivoting and movement of mandibular condyle (curved arrows). Relocation of mandibular condyle into fossa (thick black arrow).

**Image 2:**
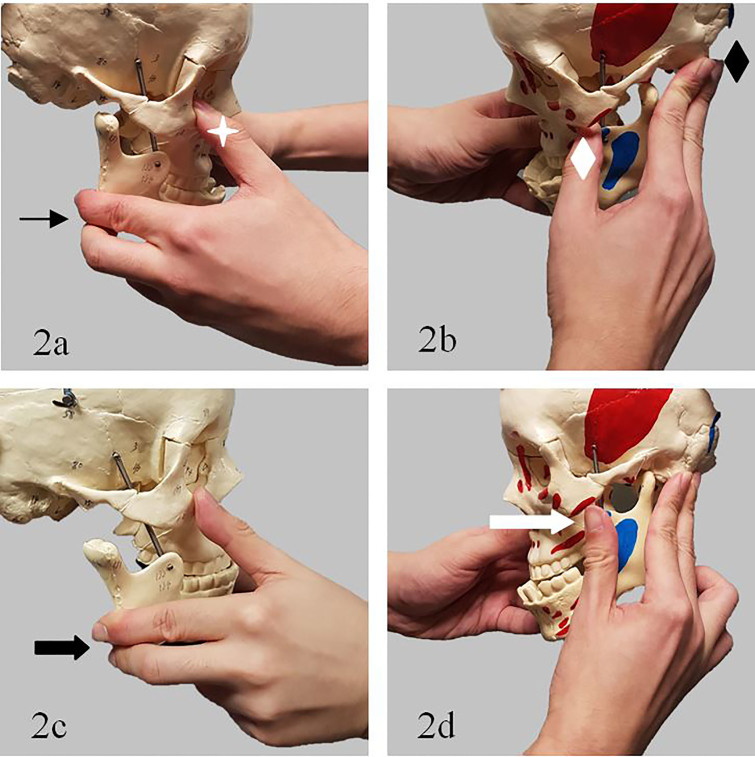
Extra-oral approach. **2a**. Thumb on malar eminence of maxilla (white star), fingers around angle of mandible (thin black arrow). **2b.** Thumb on coronoid process of contralateral side (white diamond), fingers on mastoid process (black diamond) **2c.** Fingers pull angle of mandible anteriorly (thick black arrow) **2d.** while thumb pushes coronoid process posteriorly (thick white arrow).
